# Automated generation of heuristics for biological sequence comparison

**DOI:** 10.1186/1471-2105-6-31

**Published:** 2005-02-15

**Authors:** Guy St C Slater, Ewan Birney

**Affiliations:** 1 The Ensembl Group, EMBL – European Bioinformatics Institute, Wellcome Trust Genome Campus, Hinxton, Cambridge, CB10 1SD, UK

## Abstract

**Background:**

Exhaustive methods of sequence alignment are accurate but slow, whereas heuristic approaches run quickly, but their complexity makes them more difficult to implement. We introduce bounded sparse dynamic programming (BSDP) to allow rapid approximation to exhaustive alignment. This is used within a framework whereby the alignment algorithms are described in terms of their underlying model, to allow automated development of efficient heuristic implementations which may be applied to a general set of sequence comparison problems.

**Results:**

The speed and accuracy of this approach compares favourably with existing methods. Examples of its use in the context of genome annotation are given.

**Conclusions:**

This system allows rapid implementation of heuristics approximating to many complex alignment models, and has been incorporated into the freely available sequence alignment program, exonerate.

## Background

George Box [1] once remarked that,

"All models are wrong, but some are useful."

This statement bears much relevance to biological sequence alignment, where there is no guarantee that the alignment model will accurately represent the evolutionary (or other) processes which separated the two sequences. All that is certain is that exhaustive dynamic programming (DP) algorithms (such as Smith-Waterman [2]) will yield the optimally scoring path in terms of the given model. Heuristics for sequence comparison (such as BLAST [3]) generate alignments which are valid paths through the underlying alignment model, but are not guaranteed to be optimal. Alignments generated by these heuristics can be calculated much more rapidly, and often closely match alignments which would be generated by exhaustive methods. Furthermore, many problems in the context of genome analysis consist simply of alignment of gene products (cDNA or protein) back onto the gene from which they were produced, and consequently do not require very sensitive alignment. Hence the aim here is not to attempt to develop models which are correct, but to facilitate development of models which are more *useful *in the context of large scale analyses.

Transformation between a finite state automata describing an alignment model and the recurrence relations used in DP is a powerful technique [4,5] as it allows modification of the alignment algorithm by direct manipulation of the alignment model. The Dynamite compiler [6] allows automated implementation of alignment algorithms from a description of the alignment model. This allows development of complex models which can be used to generate accurate alignments. However, calculation of alignments using these models always requires quadratic time, which is prohibitively slow for many large scale applications. This method can be applied to any alignment problem which can be represented by a regular grammar (or the equivalent finite state machine). This includes the simple three state model required for affine gaps in the Smith-Waterman-Gotoh algorithm [2,7], but also more complex models such as that used by EST_GENOME [8], where splice site prediction is integrated into the DP, allowing alignments to include introns. Other alignment models which may be expressed by a regular grammar include those allowing non-equivalenced regions such as the ABC model [9] for improved modelling of divergent loops regions in protein alignments, and DNA Block Aligner [10] which finds co-linear conserved blocks in the alignment of genomic sequences. This framework also allows models such as that used by PairWise [11] where the sequences are translated during alignment allowing for frameshifts, and GeneWise [12] which integrates translated alignments with modelling of introns for alignment of proteins against genomic DNA.

The availability of vast amounts of sequence data has generated a need for faster alignment algorithms [13]. Many of these approaches use fast algorithms to identify closely matching words which seed un-gapped alignments that are subsequently joined to form the final gapped alignment. For example, BLAST [3], FASTA [14], and sim4 [15] all operate by first finding matching words before building alignments. Such word finding can be done by a multitude of techniques, such as finite state machines used in BLAST [3], table-lookup used in SSAHA [16], or by suffix arrays as used in QUASAR [17]. Novel methods for word-based seeding of these alignments are not presented here, but rather a general system of joining these seeds to produce gapped alignments.

The alignment program FASTA [14], generates alignments from sets of seeds by performing DP confined to a diagonal band surrounding the initial matches [18]. In contrast, when building alignments, Gapped BLAST [19] permits gapped extension which allows the DP to be applied to an arbitrary high-scoring region surrounding the HSP seeds. Such heuristics allow very fast sequence alignment, but it is difficult to apply to more complex models. Furthermore, features such as introns cannot easily be incorporated into the resulting alignments without necessitating a large amount of DP to ensure that both very short exons and large introns can be included in alignments.

The aim here is to combine the strengths of Gapped BLAST and Dynamite, in a system which allows *automated generation of heuristics in terms of the underlying model. *This allows the development of heuristics for complex models such as those used in GeneWise.

Firstly, we describe bounded sparse dynamic programming (BSDP), a novel heuristic for sequence alignment, then we describe the system for automated implementation of the complex alignment algorithms required. We present proof of principle results, but this paper is primarily focussed on developing a framework for the general case.

## Implementation

The basic strategy of seeding alignments used here is the same as for BLAST, in that alignments seeds are generated, and then extended to form High-scoring Segment Pairs (HSPs), which are then joined together to form the alignments. The alignments are seeded using an Aho and Corasick [20] type finite state machine (FSM) built using the word neighbourhood of the query sequence. This generates the seeds which are extended to form the HSPs. For large scale analyses, the FSM is multiplexed using word neighbourhoods from multiple sequences. This allows analysis of multiple queries during a single pass of a genomic database, in a manner similar to that used in MPBLAST [21].

However the methods for seeding HSPs are independent from those used for building the alignments, and this paper only deals with algorithms involved in the generation of gapped alignments from sets of HSPs, and not in the calculation of the HSPs themselves.

The following strategies are employed to enable alignments to be built efficiently from sets of HSPs:

• To connect the underlying alignment model to the heuristics, a *portal *describes a set of states in the model which correspond to a set of HSPs, a *span *refers to a looping state for large alignment features such as introns, and a *SAR (Sub-Alignment Region) *describes a rectangular region on an HSP to which DP is applied.

• To avoid DP in every SAR, *upper bounds *are generated for the best alignment score for each SAR, and *BSDP (Bounded Sparse Dynamic Programming) *exploits these bounds to yield alignments in an efficient manner.

• To perform various types of DP in these SARs, the required models are generated automati cally, including C code to produce an efficient viterbi implementation for each model.

### Bounded sparse dynamic programming

Dynamic programming (for any alignment model which can be represented by a regular grammar) requires quadratic time, and hence is the most computationally expensive part of building an alignment. For pairs of sequences more than a few kilobases long, DP becomes prohibitively slow. The approach used here is similar to *sparse dynamic programming *[22], and the fragment chaining approaches used in the program sim2 [23], in that DP is applied to rectangular regions surrounding alignment seeds. However, there are two major differences in our approach. Firstly, DP is only applied to two small discrete regions on each HSP, as it is assumed that most of the HSP itself should appear in the alignment. These *sub-alignments *improve the quality of the overall alignments, and they allow complex alignment models, and large gaps such as introns to be integrated into a sparse DP framework. Secondly, as it would take too long to apply DP to every sub-alignment region (SAR), upper bounds are calculated for the DP scores for each of these SARs. This allows the sub-alignment DP to be avoided in cases where it joins HSPs which cannot feature in the final alignment, so that alignments can still be generated very rapidly even when large numbers of HSPs and SARs are involved.

**Figure 1:**
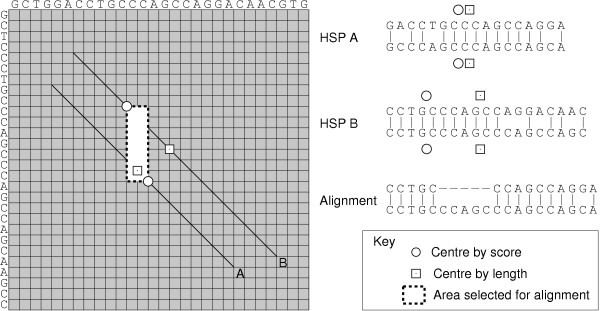
Example of joining two HSPs. A single point on each HSP is chosen to be be included in the alignment. This is the centre of the HSP according to the scores, such that equivalenced bases on either side contribute to half of the HSP score. As shown in this example, if the centre of the HSPs according to their length had been chosen instead, it would not have been possible to join them to form an alignment.

Before the BSDP can be performed, a single point on each HSP is selected which will feature in any alignment generated using that HSP. This point corresponds to a pair of equivalenced symbols which must feature in any alignment to include that HSP. A point is chosen where half the HSP score is generated by equivalenced symbols on either side of it, as this is likely to be in the highest quality portion of the HSP. As shown in the example in Figure 1, this strategy is particularly beneficial where one end of the HSP has a much lower quality than the other.

**Figure 2:**
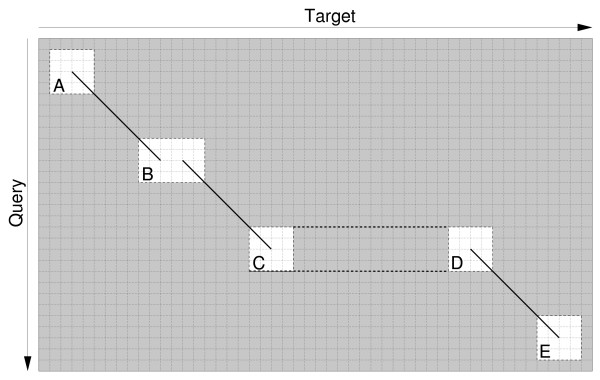
The five types of sub-alignment region around HSPs in which dynamic programming may be applied. The start of an HSP (A), handling small gaps by joining two adjacent ends of HSPs (B), handling large gaps by joining two distant HSPs via a span (C,D) and the end of an HSP (E). Other features, such as splice sites are incorporated into the alignment within the SARs.

The five types of region used for sub-alignments are classified in Figure 2. Each of these require a slightly different alignment algorithm. The alignment path must meet corners of the SARs that contain an HSP, so that the sub-alignments can be integrated with the HSPs to produce the final alignment. This approach has been primarily designed for local models, but BSDP may also be used for global and semi-global models, in which case constraints are added such that both the terminal regions (cases A and E in Figure 2) and the resulting sub-alignments must contain the relevant sequence ends. In the case of C and D, the two HSPs and their SARs are separated by a span, allowing large gaps or introns in the alignment. In these cases DP must be applied to the SAR before the span, and the end state scores must be integrated in an intermediate matrix before being provided as start state scores for the DP in the SAR after the span.

Regions for the sub-alignments are selected within the area between the centre points of the two HSPs to be joined, or in the case of terminal HSPs, between the HSP and the ends of the sequences. In addition, the positions of the SARs must be constrained to limit their size, and so that the HSPs correctly intersect with the corners of the SAR. In the case of overlapping HSPs, where there is a choice of positions for placement of the SAR, the position is chosen such that the highest scoring parts of the HSPs are outside the SARs.

**Figure 3:**
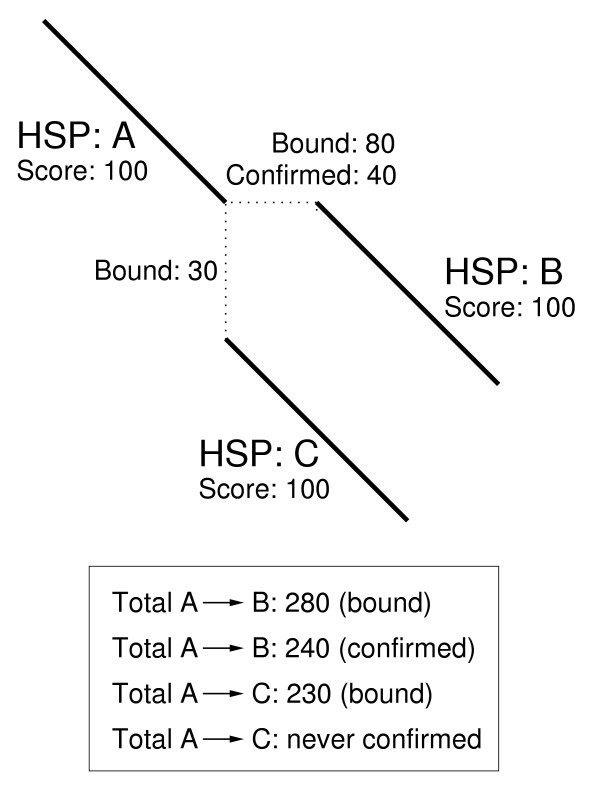
A toy example of bounded sparse dynamic programming. In this example the bounds indicate that if the overall alignment threshold is greater than 280, no sub-alignment DP is required. Otherwise, the region between A and B is confirmed first, and the bounds obviate DP between A and C. For clarity, terminal bounds are not included in this example.

Once the SARs have been selected, an upper bound is placed on the score for each sub-alignment. The calculation of these bounds is described in a later section describing automation of this method. The BSDP approach can then utilise these upper bounds to avoid application of DP in some SARs, as demonstrated by the example in Figure 3. In the case of a real alignment, a much greater number of HSPs will be involved, so the amount of DP avoided will be larger.

The BSDP is mediated through a set of priority queues, one of which is associated with each HSP, and will contain an entry for each partial alignment that ends at the HSP. The key for these priority queues is the highest score for any partial alignment ending in that HSP. Additionally, there is one global priority queue containing the highest scores from each of the other priority queues. The upper bound scores are confirmed by DP in the SARs in the current highest scoring putative alignment path. The highest scoring path will change as the scores are updated during this process. DP is applied to SARs in this way until the highest scoring path does not contain any bound scores, and then the alignment may be extracted. Upper bounds dictate that there can be no better alignment using these HSPs.

This algorithm is similar to A* search, (but differs in that many different points may be the start or end of the search), and retains the *admissibility *property of A* search, such that the result of the BSDP computation is guaranteed to be the same as if DP had been performed on every candidate SAR prior to calculation of the alignment. This is because no alignment can be extracted until all the alternative sub-alignments (which have upper bounds that indicate they could contribute to a higher-score) have been eliminated.

#### Suboptimal alignments

The BSDP alignment process can be iterated to generate sub-optimal alignments similar to those generated by the Waterman-Eggert algorithm [24], with only minimal recalculation of the partial alignments in the SARs. Each HSP may only appear in a single alignment, but further constraints are required to prevent overlapping alignments arising from overlapping SARs. The likelihood of this is occurring is greatly increased during translated alignments when HSPs in different reading frames may overlap each other.

After the first alignment has been reported, the scores for any SARs which have already been confirmed by DP, but which are not yet included in a reported alignment, are then considered as an upper bound. SARs are disallowed before recalculation when the region between the centre of the HSP and its SARs overlaps with a previously reported HSP, in which case, the SARs are disallowed. Otherwise the DP is recomputed for SARs which contain part of an alignment which has been reported since the DP for the SAR was last calculated.

### Automated model generation

As illustrated in the previous section, BSDP becomes quite complex and requires a large number of DP algorithms for computation of the alignment through the SARs. We have build a system to facilitate implementation of these models and the integration of the sub-alignments which they produce. To allow generalisation of the BSDP, everything must be defined in terms of the underlying alignment models. The alignment models are described as finite state machines, consisting of states and transitions, similar to those used in Dynamite [6].

Briefly, to convert these models into DP implementations, each state must correspond to a score in each cell of the DP matrix, and the scores for each cell are calculated by taking the maximum of the score from transitions arriving at each cell. In addition, a topological sort is required to satisfy dependency ordering for silent states.

However, in addition to automated generation of code from alignment models, the generation of the models required for DP in the SARs is also automated, as described below.

#### Building simple models

Table 1 shows a few example alignment models which are generated by this system. The models are built in a modular fashion, allowing reuse of common components such as intron models and gap models. These models may be used for exhaustive alignment in quadratic time, but in order to use them for heuristic alignment, manipulations of the models are necessary to perform DP in the SARs, as detailed in the following sections.

#### Building the heuristic model

To enable DP in the SARs for the BSDP, a heuristic model is generated from the original alignment model. This model is not used directly for calculation of alignments, but a derived model is generated corresponding to each transition in the heuristic model. Each of these derived models correspond to a type of SAR used in the BSDP.

The model is first annotated, labelling certain states as either *portal states *or *span states. *A *portal *defines a group of states which can share a set of HSPs (High-scoring Segment Pairs); these are the match states. A *span *is a state which has sequence independent looping transitions (*e.g. *states for introns, or non-equivalenced regions).

**Figure 4:**
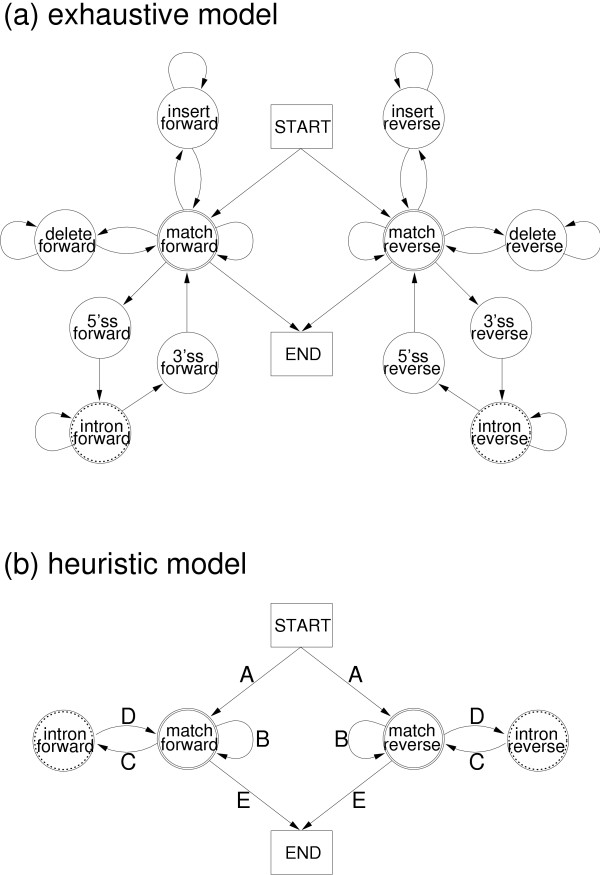
Models for alignment of an EST to genomic sequnce. The exhaustive model for aligning ESTs to genome DNA, showing portal states and span states is shown in Figure 4 (a). The portal states are *match forward *and *match reverse*, and these share a set of HSPs. The span states are *intron forward *and *intron reverse*, and may accommodate large gaps. These portal and span states are represented in the heuristic model shown in Figure 4 (b), with each transition represented by a different derived model, labelled A to E, corresponding to the SAR types show in Figure 2.

The heuristic model is build using states corresponding to each portal and span state, with transitions between these states in cases where there is a valid path between the corresponding states in the original model. An example model is shown in Figure 4 for alignment of ESTs to genomic DNA. In this example, there is a *portal *which corresponds to the *match forward *and *match reverse *states, and the *intron forward *and *intron reverse *states are *span *states.

#### Building derived models

**Figure 5:**
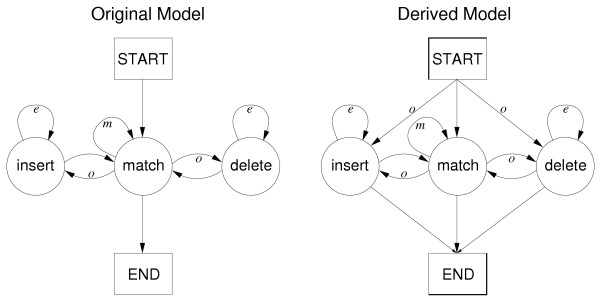
Example of generation of a derived model. The original model on the left (for Smith-Waterman-Gotoh) is used to generate the derived model on the right for joining two adjacent HSPs. Extra transitions are allowed from the START state to the INSERT and DELETE states and onto the END state, as the derived model must allow gaps to open directly from the HSPs (in contrast to Smith-Waterman-Gotoh where an alignment cannot start with a gap). Additionally, the original model is local, but in this case the derived model is global, as it must start and end on the HSP.

Derived models are produced for the DP in SARs automatically from each transition in the heuristic model. The source and destination states from each transition in the heuristic model become the start and end states in each derived model. All reachable states and transitions from these states in the original model are recursively copied to the derived model. An example of this process is shown in Figure 5. The relationships between the states and transitions in the derived model and the original model are tracked to allow conversion of the partial alignments from the derived models back to complete alignment in the original model. Terminal models (case A and E in Figure 2) are generated between portal states and a start or end state. Join models (case B in Figure 2) are generated between portal states, including from a portal state to itself. Span models (case C and D in Figure 2) are generated from a portal state to a span state, and vice versa. These allow incorporation of a large feature such as an intron into an alignment. The span models require a special end state in the model at the start of the span, and a special start start in the model at the end of a span, so that scores can be transferred from one DP matrix to the other via an intermediary score matrix.

For some cases, such as between the *match forward *and *match reverse *states, shown in Figure 4, there is no possible path, and no corresponding transition in the heuristic model, in which case, a derived model is not produced.

Ten different derived models are generated from the model shown for cDNA to genomic alignment in Figure 4, because there are two *portal *states and two *span *states in this model, and therefore, a derived model is generated for each of the five cases in Figure 2 for both the forward and reversed genes.

#### Building models for calculation of upper bounds

For each derived model, an additional model is created which is used for the pre-calculation of upper bounds for all possible sizes of SARs. For each transition in the model which has a position dependent score associated with it, the upper bound is also supplied. For example, in a match transition, the upper bound is the maximum value from the substitution matrix. A special model is created using these transition upper bounds, instead of the normal sequence-dependent transition weights. As this removes the sequence-dependent components of the algorithm, it allows pre-calculation of the upper bound for alignment score of any sequences up to the maximum permitted size of SARs. These bounds are then stored in a table for retrieval during the BSDP.

## Results

The model used in Figure 4 was used with this system in the program exonerate for rapid comparison of cDNAs and genomic DNA. This model was used to compare a test set of genomic sequences to mRNAs extracted according to the Ensembl annotations. exonerate was compared to sim4 [15], another heuristic for comparing cDNAs to genomic DNA, and EST_GENOME [8], which is implements the exhaustive DP for essentially the same underlying model. As can be seen from the results of this comparison are shown in Table 2, similar results are produced by this method, but in much less time.

This system is used for alignment of ESTs within the Ensembl gene-building pipeline [25], and a prototype implementation of this system has been used in comparison mouse and human sequences [26]. In this analysis, 13 million raw shotgun mouse reads were aligned as part of the comparative analysis of chromosome 20.

**Figure 6:**
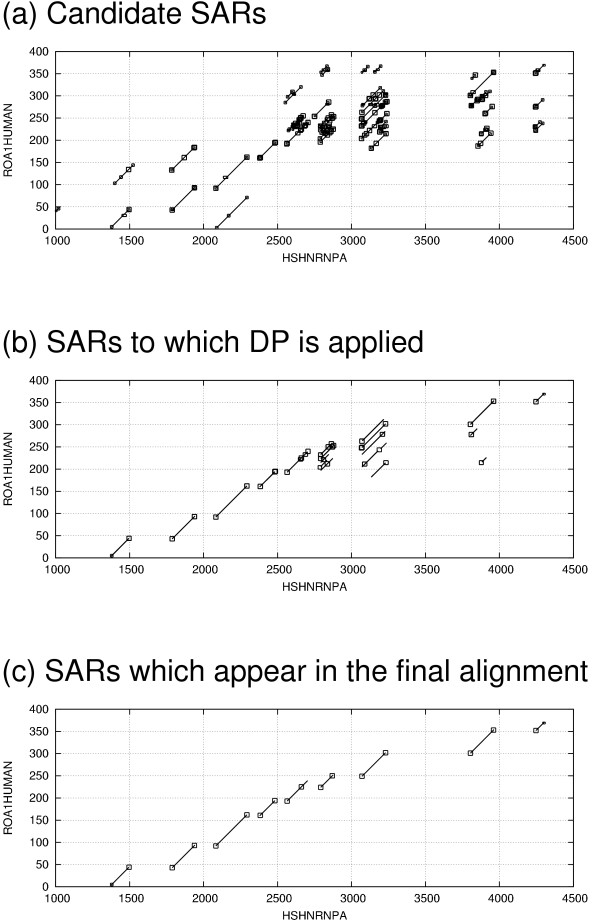
Example of BSDP during alignment of a protein sequence to genomic DNA. Figure 6 (a) shows 912 candidate SARs positioned on the HSPs. Figure 6 (b) shows the 109 SARs to which DP is applied during BSDP, and Figure 6 (c) shows the 18 SARs which appear in the final alignment.

Figure 6 shows an example the SARs generated by BSDP. Only a small proportion of these SARs will require confirmation by DP. This approach scales well – in a larger example using Titin, 27346 candidate SARs are generated, of which only 236 are confirmed and 206 appear in the final alignment. In many cases, BSDP obviates the use of DP altogether as there will be no candidate alignment with a total upper bound score over the threshold. This is especially likely to be true in cases when a high score threshold is used, such as searching for an alignment covering a certain percentage of the query, or when a dynamic score threshold is used in the search for the best few hits from a particular query to a database. Such dynamic thresholds can actually cause the search to speed up as it progresses, once good matches to the query have been found.

Another example is the alignment of the Collagen alpha 1(IX) precursor to a region of chromosome 6 containing the corresponding gene. This is a large gene, containing 38 exons over about a 90 Kb region of the genome. Using GeneWise, the alignnment required 4260 seconds (about 1 hour, 11 minutes) using exonerate to perform full DP alignment required 2700 seconds (about 45 minutes), and using exonerate with the heuristic BSDP approach required only 7 seconds, with all three methods generating identical alignments.

## Conclusion

This approach represents an advance from previous methods for automatic implementation of sequence alignment algorithms [4-6], in that it is not just the generation of code from the models which is automated, but also the generation of many of the models themselves.

This has allowed development of heuristics with sub-quadratic running times. This makes their application practical to a much larger set of problems, while retaining the much of the simplicity of their implementation.

We recognise that this approach is limited to the subset of sequence comparison problems which can be represented by a regular grammar. For example, it is not possible to use this system to model stochastic context free grammars as used in some types of RNA secondary structure such as pseudo-knots [27]. This approach is also unsuitable for the types of DP algorithms used for determining optimal segmentation in gene finding algorithms [28], however for these types of problems running time of the algorithm is not an issue.

This framework has already been used for many different alignment models ranging from simple models such as Smith-Waterman-Gotoh, to more complex models such as those for protein to genome alignment as used by GeneWise [12]. It is also well suited to problems requiring comparison of very long sequences, and we are currently extending this system to accommodate syntenic pairwise comparisons of genomic sequences of the type tackled by MUMmer [29].

Although this method is useful in many cases, for some types of distantly related sequences, the method can breakdown. For example, when long regions of the correct alignment contain many gaps with intervening sequences too short to be an alignment seed, the alignment cannot be extended beyond the boundary of the SAR, and the correct alignment may be missed. These cases can be avoided by increasing the size of the SARs, but this results in higher bound scores, so DP become necessary in more of the SARs. Such gap rich alignment are the type where the gapped HSP extension used by Gapped BLAST excels, however such gapped extension necessitates DP surrounding all HSPs, which becomes time consuming when allowing both short exons and long introns during alignments of cDNAs or proteins to genomic sequences.

As this system separates the development of the underlying alignment model from the heuristics which are built on top of them, we expect that this framework will prove useful for evaluation of the quality of the heuristics, as comparison between the alignments from the two techniques can be automated, and training may be performed to select optimal parameter sets.

## Availability

The implementation of the system described here is called C4 (to reflect the aim of producing something more powerful than Dynamite [6]), and is implemented in C programming language. It is available as part of the exonerate sequence alignment package available from , and in the exonerate module of the Ensembl CVS repository from . It is built using the glib portabilility library available from  Both exonerate and glib are available under the GNU lesser general public license. The code has been tested extensively on Linux and OSF1/alpha but has been written to be portable to many UNIX systems.

Code generation for the DP is performed to exploit compile time optimisations such as loop un- rolling and and efficient handling of edge conditions in the viterbi matrix, which is particularly beneficial for the small DP calculations required by SARs. This generated code is typically about five times faster than using a generic viterbi implementation, but produces about a million lines of code for the current set of models used by exonerate, and hence compilation takes longer.

## Figures and Tables

**Table 1:** Examples of hierarchical model building in C4. Instead of building each model from scratch, most models are created in a modular fashion by making adaptions and additions to other models. This facilitates the building of complex models.

**Model**	**Components**	**States**	**Transitions**
ungapped	substitution matrix	3	3
Needleman-Wunsch (NW)	ungapped with gap penalties	3	5
Smith-Waterman (SW)	NW + local alignment scope	3	5
Smith-Waterman-Gotoh (SWG)	SW + affine gap costs	5	9
est2genome	SWG + intron models	10	24
protein2dna	SWG + translation + frameshifts	6	13
protein2genome	p2d + intron model	13	33

**Table 2:** Results of an evaluation comparing exonerate (using the methods presented here), with sim4 and est2genome. A subset of the ensembl database was used, containing 1,827 genes, 9,917 exons and 2,387,971 bases. Times are given for alignment of all cDNAs to all contigs. The CPU time for est2genome was estimated from a smaller number of alignments. The accuracies are comparable, but the times are much faster for the heuristic methods.

**Method**	**Base Accuracy**	**Exon Accuracy**	**CPU Time (seconds)**	**x-fold**
est2genome	99.99% (2,387,648)	99.90% (9,907)	≅ 7.2 years (226,637,523)	1
sim4	98.97% (2,363,438)	98.12% (9,731)	≅ 60 hours (216,956)	1045
exonerate	99.88% (2,384,988)	99.98% (9,916)	≅ 46 minutes (2,577)	87946
